# Increased Mesostriatal Intrinsic Connectivity Associated With Cue Exposure in Adult Cannabis Users: Preliminary Findings

**DOI:** 10.1111/adb.70067

**Published:** 2025-08-07

**Authors:** Natalia M. Kleinhans, Garth E. Terry, Dahyeon Kang, Sarah F. Larsen, Mary E. Larimer

**Affiliations:** ^1^ Department of Radiology University of Washington Seattle WA USA; ^2^ Integrated Brain Imaging Center University of Washington Seattle WA USA; ^3^ Institute on Human Development and Disability University of Washington Seattle WA USA; ^4^ Department of Psychiatry and Behavioral Sciences University of Washington Seattle WA USA; ^5^ Mental Illness Research, Education, and Clinical Center VA Puget Sound Health Care System Seattle WA USA; ^6^ Department of Psychology University of Washington Seattle WA USA

**Keywords:** anticorrelation, craving, marijuana, nucleus accumbens, resting‐state fMRI, ventral tegmental area, VTA

## Abstract

Cue‐induced craving—the desire to use a drug triggered by exposure to cues associated with prior use—is a central mechanism in the development and maintenance of problematic substance use behaviours. Drug cues have the power to induce craving even in long‐term abstinent individuals, which has led clinicians to advise patients to avoid the people, places and objects that are associated with their use. This preliminary study builds on prior behavioural research that demonstrates that exposure to multimodal drug cues can increase craving even after the drug cues are removed from the environment.

We used a novel fMRI paradigm that combined multimodal cannabis cue‐exposure with resting‐state functional connectivity to examine positive and negative functional connectivity (i.e., correlations and anticorrelations) between the ventral tegmental area (VTA) and the striatum, a circuit critically involved in reward processing and addiction. Intrinsic VTA‐striatal connectivity was measured in 28 individuals who use cannabis regularly (CU group) and 26 age‐ and sex‐matched controls who had never used cannabis before and after multimodal (visual and olfactory) cannabis cue exposure. Craving was assessed at baseline using the Marijuana Craving Questionnaire‐Short Form to test whether VTA‐striatal connectivity was correlated with self‐reported craving measured prior to the fMRI scan. There were no significant group differences in VTA‐striatal connectivity during the baseline resting‐state scan. However, following cue exposure, CU participants showed significantly greater VTA‐caudate connectivity compared to controls. Further, within the CU group, baseline craving was positively correlated with VTA‐striatal connectivity at both time points.

Our preliminary findings support prior investigations demonstrating that alterations of mesostriatal connectivity are associated with cannabis use and craving in individuals with problematic cannabis use. In addition, the observation of altered connectivity during the post‐cue resting‐state scan—after multimodal cannabis cues were removed—suggests a potential neural mechanism by which cue exposure may contribute to relapse vulnerability in individuals with problematic cannabis use.

## Abstract 2

We observed that resting‐state connectivity between the ventral tegmental area (VTA) and the nucleus accumbens and head of the caudate are anticorrelated. Preliminary findings show that cannabis cue‐exposure is associated with altered intrinsic connectivity between the VTA and the head of the caudate in the cannabis group after cannabis cues are no longer present. Further, within the cannabis group, craving measured prior to scanning was positively correlated with VTA‐striatal connectivity at both time points, independent of cannabis cue‐exposure.

## Graphical abstract

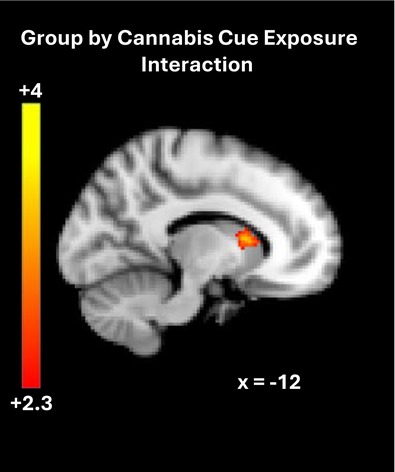

**Abbreviations**
ASSIST: Alcohol, Smoking, and Substance Involvement Test
AUDIT: Alcohol Use Disorder Identification Test
CUDIT‐R: Cannabis Use Disorder Identification Test Revised
FEAT: FMRI Expert Analysis Tool
FLAME: FMRIB's Local Analysis of Mixed Effects
MCQ‐SF: Marijuana Craving Questionnaire‐Short Form
MNI: Montreal Neurological Institute

## 1 Introduction

Cue‐induced craving—the desire to use a drug triggered by exposure to cues associated with prior use—is a central mechanism in the development and maintenance of problematic substance use behaviours. It is characterized by an intense desire to self‐administer a drug and can lead to relapse after treatment or during abstinence [1, 2, 3, 4].

To better understand the underlying mechanisms of cue‐induced craving, laboratory studies have developed paradigms to elicit cravings and explore the mechanisms underlying the drug craving experience. Numerous cue‐reactivity studies, employing a variety of methodologies—including self‐reports, behavioural measures, electroencephalography (EEG) and functional magnetic resonance imaging (fMRI)—have advanced our understanding of the role of craving in addiction [5, 6, 7, 8]. Although only a limited amount of research has been conducted using cannabis cues, cue‐reactivity has been found to predict treatment outcomes and relapse in cigarette, alcohol and heroin addiction [9, 10, 11], suggesting that it may also play a role in the development and maintenance of other substance use disorders, including cannabis use disorder (CUD). Among the methodologies employed in cue‐reactivity research, fMRI has been thought to be particularly valuable for its ability to measure brain activity with high spatial resolution, providing insights into the neural mechanisms of cannabis craving in humans. Typical fMRI cue‐reactivity paradigms employ task‐based designs that alternate between presenting drug‐related and control (neutral or non‐drug) cues. Hemodynamic responses to these cues are contrasted to identify regions exhibiting greater activation to drug cues, thereby revealing neural substrates of craving and cue reactivity [12, 13, 14]. Previous fMRI cue reactivity studies have shown increased activation in response to visual cannabis cues in the ventral tegmental area (VTA), anterior cingulate cortex (ACC), orbital frontal cortex (OFC), striatum, insula, cerebellum, thalamus, pre‐ and post‐central gyri, inferior parietal lobe and superior temporal gyrus [15, 16], and increased functional connectivity during cannabis cue‐reactivity tasks between the nucleus accumbens and the amygdala and anterior cingulate in cannabis‐dependent individuals compared to non‐dependent cannabis‐using individuals [17]. In addition, activation in the occipital cortex, hippocampus, superior temporal pole and middle occipital gyrus has all been positively correlated with subjective reports of cannabis craving [18], suggesting these regions may be sensitive to individual differences in addiction severity.

Traditional task‐based cue‐reactivity paradigms may not fully capture the prolonged nature of craving responses. Prior behavioural research has demonstrated cue‐induced subjective craving (desire and urge to use cannabis) persists up to 150 min post‐cue exposure, and physiological responses (i.e., increase in diastolic blood pressure) can last up to 15 min post cue [19]. These findings may suggest a key limitation of traditional cue‐reactivity fMRI designs, which typically alternate between substance‐related and neutral or non‐substance control cues quickly with intervals ranging from a few seconds [20, 21] to less than a minute [22, 23]. Such brief intervals may fail to capture the prolonged craving response and are susceptible to carryover effects, potentially reducing the power and interpretability of task‐based fMRI studies. Given that craving‐related neurobiological responses may continue well beyond the duration of the cue presentation itself, resting‐state scans conducted before and after cue exposure offer a complementary approach to capture more sustained, intrinsic brain activity related to a change in the physiological state of a participant following cue exposure. To address this, we designed a novel resting‐state cue reactivity paradigm that measures intrinsic connectivity both prior to and after exposure to multimodal cannabis cues, enabling the examination of prolonged neurobiological reactivity. This design allowed us to test brain activation using a well‐established task‐based approach (see [24]) as well as testing our novel approach intended to measure whether mesostriatal brain networks evidence prolonged connectivity changes following the removal of cannabis cue exposure.

This approach aligns with and extends prior work on resting‐state functional connectivity in cannabis users. A systematic review of resting‐state functional connectivity studies showed that heavy cannabis users or those with CUD have increased functional connectivity of the default mode, salience attention, central executive and striatal networks [25]. However, connectivity with the VTA, the main source of dopamine to reward circuitry and a theoretically critical component of the neurobiology of addiction, has only been reported by one group [26]. This study found increased local hyperconnectivity density in the midbrain, ventral striatum, brain stem and lateral thalamus during a resting state fMRI scan, but no difference in long‐distance VTA connectivity in individuals with cannabis dependence. Interestingly, studies probing the dopaminergic system with positron emission tomography demonstrate that cannabis users and those with CUD have reduced dopamine synthesis capacity in the striatum, may have blunted striatal dopamine release following pharmacological stimulant or stress challenge, yet have no difference in dopamine D_2_/D_3_ receptor availability in the striatum [27]. The goal of our study was to expand upon previous work to determine whether cue exposure in regular cannabis users modulates intrinsic connectivity between the VTA and the striatum, a dopaminergic pathway involved in reward processing and habit formation. In addition, we investigated how connectivity between the VTA and striatum is related to self‐reported measures of craving in individuals at risk for CUD vs. cannabis‐naïve individuals.

We hypothesized that cannabis users would exhibit increased connectivity between the VTA and striatum, particularly the nucleus accumbens, due to prior findings of striatal hyperconnectivity in cannabis users and theoretical models implicating dopaminergic sensitization in cue‐induced craving and addiction‐related behaviours. Although only a limited number of studies have examined VTA‐based functional connectivity in this population, prior research suggests that dopaminergic circuits are sensitive to substance‐related cues even in non‐dependent or subclinical samples [8]. Given that our sample consisted of individuals who regularly used cannabis, we anticipated that cue exposure would elicit altered intrinsic VTA‐striatal connectivity, similar to what has been observed in individuals with diagnosed substance use disorders. In addition, we expected that higher levels of self‐reported craving would be associated with greater VTA‐striatal connectivity across participants, consistent with the proposed role of this circuit in motivational salience and conditioned drug responses. Finally, we hypothesized a group‐by‐timepoint interaction, such that exposure to multimodal cannabis cues would result in greater post‐cue increases in VTA–nucleus accumbens connectivity in the cannabis group relative to controls, reflecting sustained, heightened neurobiological responsivity to drug‐related stimuli.

## 2 Methods

### 2.1 Participants

Participants (*N* = 54; 28 cannabis use [CU], 26 controls) were recruited from the Seattle metropolitan area and were required to be between 21 and 45 years of age to be eligible for the study. Inclusion in the CU group was determined using the Cannabis sub‐test of the Alcohol, Smoking and Substance Involvement Screening Test (ASSIST) [28]; participants who qualified as moderate to high risk for a CUD (ASSIST Substance Involvement Score ≥ 4) and also reported weekly to daily CU in the prior year were enrolled in the CU group. Inclusion in the control group was based on self‐report of no lifetime history of CU. Participants in both groups were additionally screened using a semi‐structured interview and excluded for the following: received a diagnosis of or treatment for schizophrenia or other psychotic disorder, bipolar disorder or depression within the past 6 months; reported high risk alcohol use (CAGE score > 2), or reported moderate to high risk use of other illicit substances (ASSIST Substance Involvement Score ≥ 4 for each substance reported, e.g., inhalants, cocaine); or reported current psychotropic medication, significant neurological medical history, diagnosis of hyposmia, MRI contraindications or left‐handedness. Five participants (three CU, two controls) were excluded from analyses due to fMRI data quality issues (*n* = 2), a technical difficulty with the cannabis cue reactivity task that invalidated the second resting state scan (*n* = 1), and initiating psychotropic medication use following enrolment (*n* = 2).

This study was approved by the University of Washington Human Subjects Institutional Review Board. Informed written consent was obtained from all study participants. Participants were paid $50 for attending the research visit.

### 2.2 Procedures

All participants were asked to refrain from using cannabis for at least 48 h prior to the research visit. Three CU participants abstained from CU for less than 48 h (34.35 h, 46.06 h, 47.60 h). The average amount of time between last CU and the research visit was 4.1 days (SD = 3.39).

### 2.3 Assessment of Cannabis, Alcohol, and Tobacco Use

Prior to entering the scanner, participants were screened to ensure they did not have upper respiratory symptoms that would impact their ability to smell. Then, before entering the scanner at the research visit, they completed questionnaires including the Marijuana Craving Questionnaire—Short Form (MCQ—SF) [11], a twelve‐item questionnaire covering compulsivity, emotionality, expectancy and purposefulness; the Cannabis Use Disorder Identification Test Revised (CUDIT‐R) [29]; the Alcohol Use Identification Test (AUDIT) [30]; the ASSIST for Tobacco use; and the Quantification of Cannabis Consumption, a semi‐structured interview used to measure current self‐reported CU (see Supplementary information). Information gathered included type of cannabis used, age of first use/regular use, current use levels and history of withdrawal‐related symptoms when stopping or reducing CU, including physical symptoms (e.g., shakiness, abdominal pain, sweatiness or headache), mood symptoms (e.g., irritability, anxiety or depressed mood), sleep problems and changes in appetite. See Table 1.

**TABLE 1:** Participant characteristics.

	CUD (*n* = 25)	Control (*n* = 24)	*t* (df)	*p*
Sex (Male:Female)	13:12	13:11		
Race				
Caucasian	21	11		
Asian	1	11		
African American	1	0		
Other	2	2		
Ethnicity				
Hispanic/Latino	3	1		
Not Hispanic/Latino	22	23		
Age (years), mean (SD)	26.17 (4.15)	26.29 (5.14)	0.08 (47)	0.933
Substance use measures, mean (SD)				
ASSIST–Cannabis	12.1 (6.20)	0 (0)	−9.53(47)	< 0.001
AUDIT	6.6 (4.33)	2.54 (2.17)	−4.12(47)	< 0.001
ASSIST–Tobacco	3.64 (5.60)	0.58 (1.93)	−2.53(47)	0.015
CUDIT‐R total	10.36(5.20)	0 (0)	−9.56(47)	< 0.001
MCQ‐SF total	35.16 (9.13)	17.29 (9.66)	−6.65(47)	< 0.001
Age at first use of cannabis (years)	17.32 (−4.66)	NA		
Duration of at least weekly use of cannabis (years)	4.12 (−3.38)	NA		
Mode of cannabis consumption, *n* (%)				
Cannabis flower	24 (96)	NA		
Extract (concentrated oil, wax, etc.)	9 (36)	NA		
Edibles	14 (56)	NA		
Hash	3 (12)	NA		
Frequency of cannabis use, *n* (%)				
4+ times per day	1 (4)	NA		
1–2 times per day	6 (24)	NA		
4+ times per week	5 (20)	NA		
2–3 times per week	9 (36)	NA		
2–4 per month	4 (16)	NA		

Abbreviations: ASSIST = Alcohol, Smoking and Substance Involvement Screening Test; AUDIT = Alcohol Use Disorder Identification Test; CUDIT‐R = Cannabis Use Disorder Identification Test – Revised; NA = not applicable; SD = standard deviation.

### 2.4 Resting State fMRI Protocol

**FIGURE 1:**
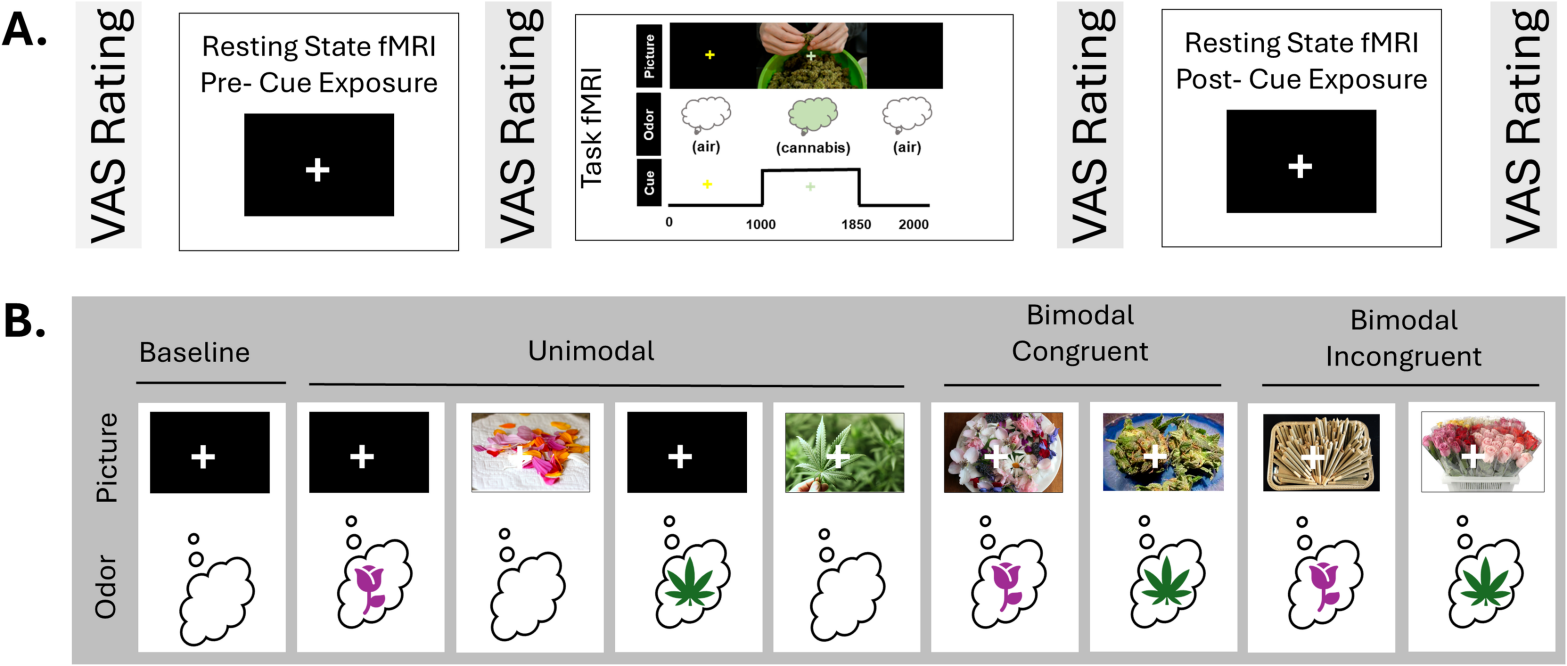
(A) Experimental design consisting of two resting state fMRI scans separated by a 12‐min event related fMRI cue‐reactivity task. The VAS rating scale was administered prior to each fMRI scan. During each cue‐exposure fMRI trial, participants were instructed to prepare to breathe when they saw the yellow cross, and to inhale when the green cross appeared. This figure depicts an example of a bimodal‐congruent trial. (B) Example cannabis and control cue stimuli presented in the fMRI cue‐reactivity task, grouped by type: unimodal, bimodal‐congruent (picture and odour match), bimodal‐incongruent (picture and odour differ). Pink roses = phenylethyl alcohol odorant; green cannabis leaves = cannabis odorant.

Resting state connectivity was investigated before and after cannabis cue exposure. During the resting state scans, participants were instructed to stare at the cross, relax, let their minds wander and to make sure not to fall asleep. In between the two resting‐state scans, participants were exposed to cannabis cues via a 12‐min event‐related fMRI cue‐reactivity paradigm (see [24] for details). During the cue exposure portion of this experiment, participants were randomly exposed to 66 pictures of cannabis products/paraphernalia or non‐psychoactive flowers and plants presented for 850 ms at a time and 66 odour exposures for 850 ms to the cannabis odorant Cannaroma, created by The Werc Shop, based on the terpene profile of the cannabis strain ‘Blue Dream’, or the odorant phenylethyl alcohol, which smells like roses. Participants were instructed to prepare to breathe in when they saw a yellow cross‐hair and to inhale through their nose when the green crosshair appeared (see Figure 1). They were informed that sometimes they would be able to smell a flowery smell, sometimes they would smell a cannabis‐like smell, and sometimes they would smell neither. Participants were informed before the cue exposure task that there was no THC in the cannabis odorant. Stimulus types were unimodal (picture or odour), bimodal—congruent (e.g., picture of a joint combined with the cannabis odorant) or bimodal—incongruent (e.g., picture of roses combined with the cannabis odorant) (see Figure 1 for examples).

Participants rated their current craving levels at four points during the experimental session: prior to the first resting‐state scan, before and after the cue‐reactivity fMRI task and immediately following the second resting‐state scan. At each time point, participants were administered three Visual Analog Scale (VAS) items over the intercom and asked to rate their agreement with each statement on a scale from 1 (not at all) to 10 (extremely). The statements were as follows: (1) “I crave marijuana right now,” (2) “I want to use marijuana right now” and (3) “Marijuana sounds very appealing to me right now.”

### 2.5 Structural and Functional MR Image Acquisition

Structural and functional MRIs were collected on a 3T Phillips Achieva MR system (version 1.5, Philips Medical System, Best, The Netherlands) using a phased array 32‐channel sensitivity encoding (SENSE) head coil. A T1‐weighted MPRAGE (magnetization prepared‐rapid gradient echo; TR 7.6 ms; TE 3.6; flip angle = 7, FOV = 176 mm, matrix 176 × 256, 176 slices, acquisition voxel size (mm) = 1.00/1.00/1.00; reconstruction voxel size = (mm) = 1.00/1.00/1.00; TFE shots = 128; TFE durations = 1963.3 ms; inversion delay (TI) 910.5 ms; slice orientation transverse, foldover direction AP; REST slab 64.2 mm slice thickness) was collected for co‐registration and anatomical localization. Resting‐state fMRI data were acquired using a single‐shot gradient‐recalled echo‐planer imaging (EPI) sequence (TR = 2000 ms; TE = 24 ms; flip angle = 79°; FOV = 240 mm) with a matrix size of 80 × 78 (in‐plane resolution = 3 × 3 mm). Thirty‐nine axial slices covering the entire brain (slice thickness = 4 mm, gap = 0 mm) were acquired during each scan using ascending slice acquisition. Two hundred volumes were collected during each resting state scan (preCue and postCue; 6′ 40″ each). Participants' heads were immobilized during the scan with padding to minimize movement.

### 2.6 fMRI Preprocessing

MRI data preprocessing was performed using FSL version 5.0 (http://www.fmrib.ox.ac.uk/fsl/) and AFNI version 16.0.11 (http://afni.nimh.nih.gov/afni/). Our preprocessing pipeline consisted of (1) brain extraction using BET, (2) simultaneous motion‐ and slice‐timing correction [31], (3) spike artefact removal using AFNI 3dDespike, (4) high‐pass filtering [sigma = 50s] and (5) spatial smoothing [FWHM = 5 mm]. Physiological noise was corrected by filtering out the mean timeseries of the signal from the ventricles. In addition, motion‐related confounds were addressed by filtering out motion parameters (framewise displacement and dvars calculated via fsl_motion_outliers) and single‐point motion regressors (volumes for which the frame‐to‐frame displacement exceeded 0.5 mm or 0.5° on any axis). To reduce the effects of head motion, participants with a mean absolute motion value (root mean square, as calculated by FSL mcflirt) greater than 1.0 for either resting state scan were excluded (*n* = 2).

The VTA seed region for intrinsic connectivity analyses was identified using the Duke University Probabilistic Atlases of the Midbrain [32], thresholded at 50% probability (see supplementary information, Figure S3, for a visual depiction of the VTA mask). The VTA atlas mask was warped into native space, and the mean signal of the voxels underlying the VTA was extracted for each participant's resting‐state scan.

### 2.7 fMRI Imaging Analyses

Individual‐level connectivity maps for the VTA were derived using FMRIB's Improved Linear Model (FILM) with local autocorrelation correction [33] for each resting state scan. Within‐subject contrasts were computed using fixed effects analyses in FMRI Expert Analysis Tool (FEAT) for the following contrasts: preCue, postCue, postCue > preCue and preCue + postCue. Both positive correlations and anti‐correlations with the VTA timeseries were tested.

Higher level analyses of VTA connectivity for positive correlations and anti‐correlations for the contrasts preCue, postCue and preCue + postCue were conducted using FLAME (FMRIB's Local Analysis of Mixed Effects) and included all study participants (*N* = 49). In addition, group means were tested for the postCue > preCue contrast. Tests of between‐group differences were conducted for each contrast (preCue, postCue, postCue > preCue and preCue + postCue). Statistical corrections for multiple comparisons were conducted using cluster‐thresholding based on Markov Chain Monte Carlo sampling (voxel height of z > 2.3) and a small volume corrected cluster significance threshold of *p* < 0.05) for the bilateral nucleus accumbens, putamen and caudate nucleus. The three striatal regions were delineated and results were labelled according to the Oxford‐GSK‐Imanova Structural–anatomical Striatal Atlas [34]. In addition, whole brain analyses were performed for exploratory purposes.

To measure correlations between VTA‐striatal connectivity and craving in the CU group, the baseline total MCQ‐SF scores were entered as regressors, controlling for age, for each contrast.

### 2.8 Statistical Analysis of Behavioural Measures

All statistical analyses were completed using SPSS Version 29. Between‐group differences were tested using independent‐sample *t*‐tests.

## 3 Results

### 3.1 Substance Use

Based on the ASSIST, 24/25 CU participants were classified as ‘moderate’ (≥ 4) and 1/25 CU participant was classified as ‘high’ (≥ 27) risk for a CUD. In addition, participants in the CU group reported significantly higher symptoms on the AUDIT (CU mean = 6.60(4.33), control mean = 2.54(2.17); *t* = −4.12, *p* < 0.001) and a significantly higher substance involvement score on ASSIST‐Tobacco (CU mean [SD] = 3.64(5.60), control mean [SD] = 0.58(1.93); *t* = −2.43, *p* < 0.05) than the control group (Table 1). No participants in either group reported a lifetime history of physical withdrawal symptoms. Other symptoms of withdrawal were reported by 13/25 CU participants: five reported mood symptoms, two reported sleep problems and four reported changes in appetite; one participant reported both mood and appetite symptoms, and another endorsed mood, sleep, and appetite symptoms.

### 3.2 Visual Analog Scale

Compared to the control group, the CU group reported significantly higher ratings at each time point, including before the first resting‐state scan, before and after the event‐related cue‐reactivity fMRI task, and after the second resting‐state scan. In the control group, mean VAS scores remained at or near the floor (mean = 1.00 across most items and timepoints), while the CU group consistently endorsed moderate levels of craving (means ranging from 3.00 to 4.50). Between‐group differences at each time point were statistically significant for all items (*p*‐values < 0.001), as shown in Table 4.

### 3.3 Motion Parameters

An independent samples *t*‐test was used to test between‐group differences in the average root mean square of the absolute motion (movement relative to the fiducial time point) across the entire run. No between‐group differences in the root mean square of the motion parameters were present for preCue resting state scan: CU group mean = 0.193 (0.075), control group mean = 0.195 (0.088); *t*(47) = 0.06, *p* = 0.953; range of motion for CU = 0.109–0.369 and for CONTROL = 0.094–0.440; or postCue resting state scan: CU group mean = 0.208 (0.093), control group mean = 0.210 (0.107); t(47) = 0.08, *p* = 0.940; range of motion for CU = 0.099–0.452 and for CONTROL = 0.111–0.573.

### 3.4 Neuroimaging Findings

#### 3.4.1 VTA‐Striatal Connectivity Maps

**FIGURE 2:**
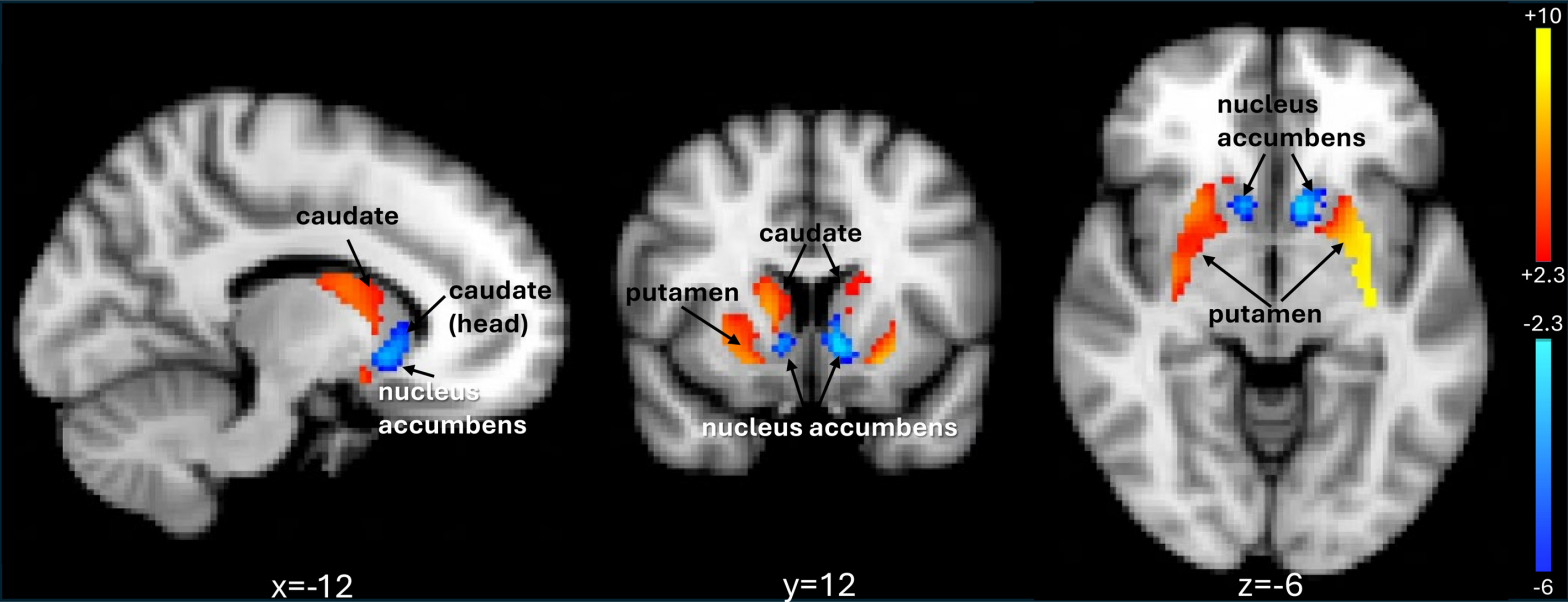
All study (*N* = 49) group intrinsic connectivity maps (preCue + postCue). The ventral tegmental area is significantly correlated with the putamen and body of the caudate (red‐yellow clusters) and anticorrelated with the nucleus accumbens and head of the caudate (blue to light blue clusters). Images are presented in radiological convention (R = L).

Activation maps depicting significant correlations and anti‐correlations with the striatum are displayed in Figure 2 and reported in Table 2. Within‐group analyses of the contrast postCue > preCue showed increased VTA‐caudate connectivity in the caudate in the CU group. No significant differences in connectivity between the pre and postCue resting state scans (postCue > preCue) were observed in the control group.

**TABLE 2:** VTA‐striatal connectivity.

Contrast		+/− corr	ROI	Voxels	*p*	z‐max	MNI (mm)		
							x	y	z
PreCue + PostCue									
	CON+CU (N = 49)	+	Right caudate	346	0.001	7.96	14	2	14
			Left caudate	199	0.006	5.79	−14	0	16
			Right putamen	649	< 0.001	8.38	28	−6	2
			Left putamen	646	< 0.001	10.3	−30	−16	−8
	CON+CU (N = 49)	−	Left nucleus acc.	85	0.012	5.56	−10	14	−6
			Right nucleus acc.	38	0.030	4.86	10	14	−6
			Left caudate	108	0.022	4.79	−18	20	0
PostCue > PreCue									
	CU	+	Left caudate	117	0.044	3.39	−14	12	10
	CU>CON	+	Left caudate	175	0.017	3.72	−18	18	6
			Right caudate	168	0.019	3.63	12	2	16

*Note:* Regions are labelled using the Oxford–Imanova Striatal Structural Atlas. *p*‐Values are based on small volume cluster correction for multiple comparisons. A sensitivity analysis excluding the CU participant with an ASSIST cannabis subscale score > 27 yielded the same significant effects reported here.

#### 3.4.2 Between Group Comparisons

There were no significant between‐group differences in VTA‐striatal connectivity during the preCue scan, postCue scan or when the preCue and postCue scans were combined (perCue + postCue).

#### 3.4.3 Interaction Between Groups and Cue Exposure

**FIGURE 3:**
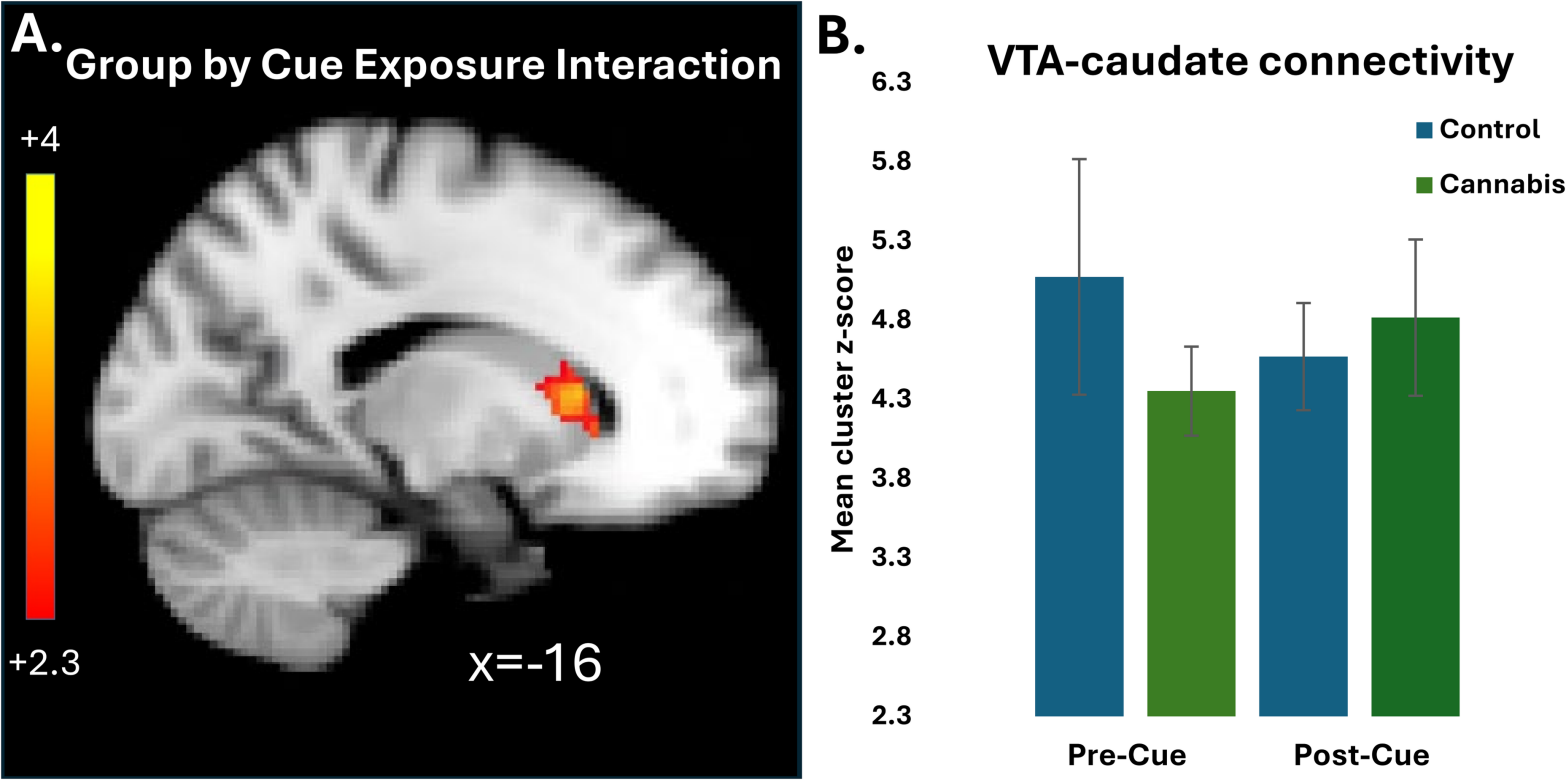
Group × time interaction in ventral tegmental area (VTA)‐striatal resting‐state connectivity. (A) Sagittal image cluster shows the significant interaction effect in VTA connectivity with the head of the caudate nucleus. (B) Bar chart illustrates the mean z‐score of the all the voxels in the caudate cluster at each time point for each group separately (controls = blue, CU = green). CU participants showed increased connectivity following cue expose between the VTA and caudate, while the control participants showed reduced connectivity in VTA connectivity between the two resting‐state scans.

There was a significant group by cue interaction effect in the head of the caudate nucleus (Table 2, Figure 3). The control participants demonstrated reduced connectivity between the pre and postCue resting state scans, while the CU group showed increased connectivity at postCue relative to the preCue resting‐state scan.

#### 3.4.4 Correlations Between Connectivity and Cannabis Craving

**FIGURE 4:**
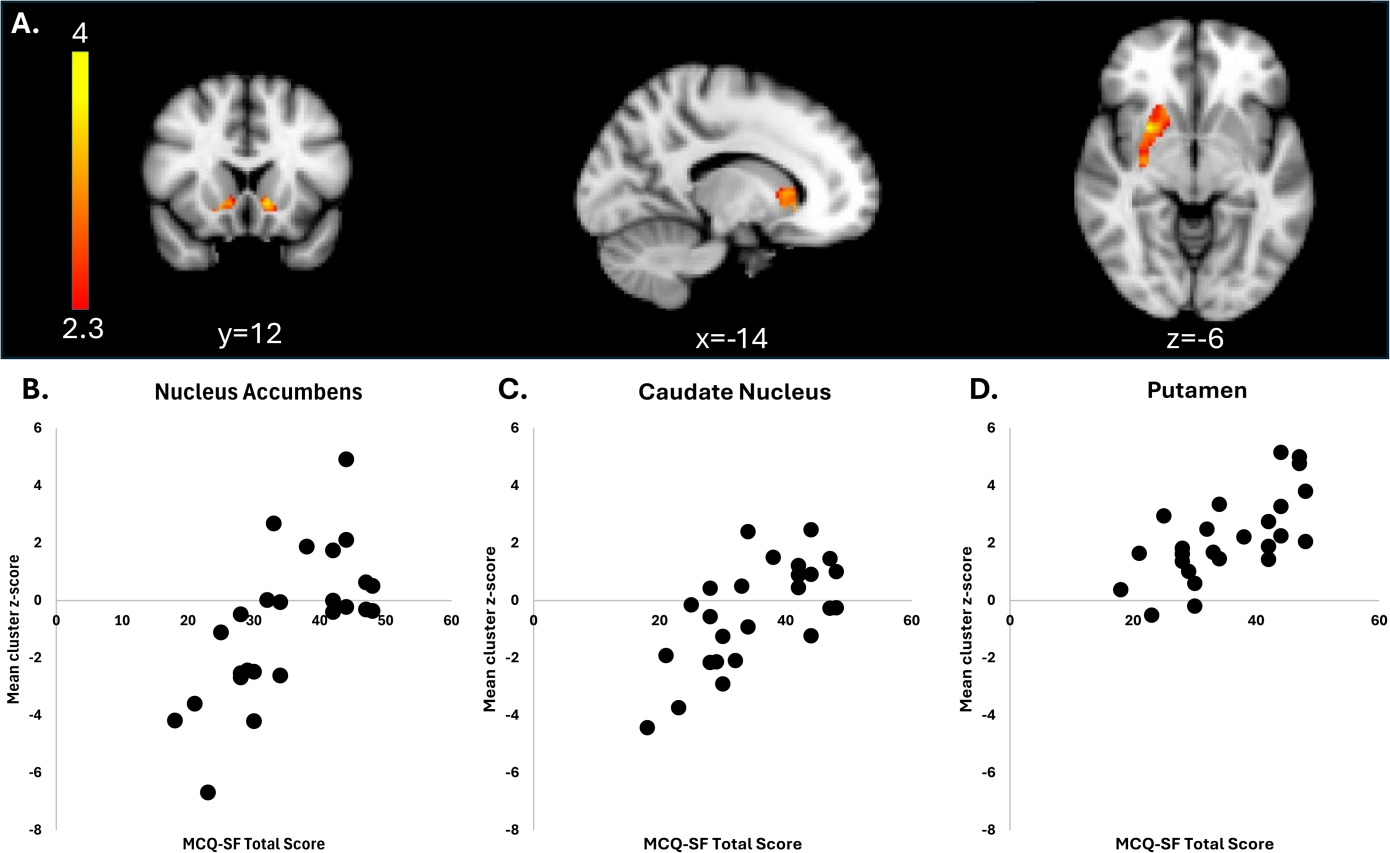
Correlations with ventral tegmental area‐striatal connectivity (preCue + postCue) and craving in the CU group. (A) Significant clusters of activation were observed in the nucleus accumbens (left), the head of the caudate (middle) and the right putamen (right). (B) Scatter plots reflect the mean z‐score within the significant cluster for each individual and are included for illustrative purposes only. Images are presented in radiological convention (R = L).

Significant, positive correlations were observed between VTA connectivity to the NA, caudate and putamen and higher self‐reported levels of craving in the CU group for all contrasts (preCue, postCue and preCue + postCue). Results for the preCue + postCue contrast are reported in Table 3 and depicted in Figure 4. No significant correlations were observed between the postCue > preCue contrast and self‐reported craving.

**TABLE 3:** Correlations between VTA connectivity and craving in the cannabis use group.

Contrast	+/− corr.	ROI	Voxels	*p*	z‐max	MNI (mm)		
						x	y	z
PreCue + PostCue	+	Right nucleus acc.	109	0.007	4.28	8	8	−2
	+	Left nucleus acc.	68	0.015	4.22	−14	20	−8
	+	Right putamen	439	0.000	4.23	26	8	−6
	+	Left caudate	142	0.011	3.61	−16	22	−4
	+	Right caudate	65	0.045	3.99	16	20	−2

*Note:* Regions were delineated using the Oxford–Imanova Striatal Structural Atlas. *p*‐Values are based on small volume cluster correction for multiple comparisons. All results remain statistically significant after excluding the cannabis use participant with an elevated ASSIST cannabis score (> 27), with the exception of the right caudate, which remained at a trend level (*p* = 0.057).

**TABLE 4:** Visual analog scale (VAS; 1–10 range) ratings of craving across experimental time points.

	CU			Control			*t*	*p* val
	Mean	SD		Mean	SD			
Pre‐Cue resting state								
I crave marijuana right now.	3.19	2.25		1.00	0.00		−4.49	< 0.001
I want to use marijuana right now.	3.42	2.37		1.00	0.00		−4.72	< 0.001
Marijuana sounds very appealing to me right now.	4.12	2.55		1.28	0.83		−4.82	< 0.001
Pre‐cue reactivity task								
I crave marijuana right now.	3.08	2.55		1.00	0.00		−4.84	< 0.001
I want to use marijuana right now.	3.62	2.07		1.00	0.00		−5.57	< 0.001
Marijuana sounds very appealing to me right now.	4.27	2.29		1.24	0.81		−4.98	< 0.001
Post‐cue reactivity task								
I crave marijuana right now.	3.50	2.86		1.04	0.20		−5.03	< 0.001
I want to use marijuana right now.	3.81	2.44		1.08	0.27		−4.82	< 0.001
Marijuana sounds very appealing to me right now.	4.50	2.82		1.28	1.00		−5.03	< 0.001
Post‐Cue resting state								
I crave marijuana right now.	3.00	3.05		1.00	0.00		−4.28	< 0.001
I want to use marijuana right now.	3.38	2.32		1.00	0.00		−4.87	< 0.001
Marijuana sounds very appealing to me right now.	4.04	2.43		1.08	0.39		−5.20	< 0.001

*Note:* Degrees of freedom for each *t*‐test = 47. CU = cannabis using group.

Results of exploratory whole brain analyses are reported in the supplementary information (Tables S1 and S2, Figure S1). In addition, results of sensitivity analyses conducted with the outlier participant who had a cannabis ASSIST score that exceeded 27 removed (Tables S3, S4), and conducted with the AUDIT as a covariate are included in supplementary information (Tables S5, S6).

## 4 Discussion

This preliminary study investigated functional connectivity within the mesostriatal reward system associated with regular CU. Our seed region was placed in the VTA, a critical node of the mesostriatal system, which is involved in releasing and modulating dopamine. The main target of dopaminergic neurons from the VTA is the striatum, and results of our cannabis cue‐reactivity study showed increased activation in the striatum and VTA in CU participants compared to controls in response to cannabis cues [24]. Notably, fMRI activation in the VTA during reward anticipation has been shown to be correlated to dopamine release [35]. Here, we demonstrated that intrinsic connectivity between the VTA and caudate nucleus increases in individuals at risk for CUD during a 10‐min window of time after exposure to cannabis cues.

We measured baseline intrinsic connectivity (following 24–48 h of abstinence from CU) and intrinsic connectivity after exposure to visual and olfactory cannabis cues. Previous research in other drugs of abuse has found that exposure to drug‐associated cues increases brain concentrations of dopamine in both the dorsal and ventral striatum among individuals with substance use disorders [36, 37] with the increase in the magnitude of this dopamine response correlating strongly with individual differences in their subjective rewarding effects. At baseline (prior to cue presentation), connectivity between the VTA and striatum did not significantly differ between cannabis users and control participants, consistent with Manza et al. [26]. However, when comparing the change in connectivity from the preCue to postCue conditions, regular cannabis users showed increased connectivity between the VTA and the head of the caudate nucleus, while control participants did not show significant differences in connectivity over time. No group differences in connectivity in response to cue exposure were observed in the nucleus accumbens or putamen. This work indicates the associative loop component of the striatum [38] plays a key role in mediating goal‐directed behaviour in response to cannabis cues in individuals at risk for CUD. The persistent coactivation of the VTA and caudate following cue exposure in cannabis users may reflect an enhanced motivational drive or increased salience of cannabis‐related cues. While our study showed this effect was limited to the caudate, it is possible that in individuals with more severe addiction, connectivity changes might extend to the putamen. Such a shift would signify engagement of the habit system, reflecting a transition from goal‐directed to habitual, automatic drug‐seeking behaviours. Future studies should explore the role of addiction severity in modulating these connectivity patterns, particularly within the dorsal striatum.

Contrary to our hypothesis, exposure to cannabis cues did not differentially impact connectivity between the VTA and nucleus accumbens in controls compared to cannabis users. Our hypothesis was based on previous studies that have shown firing rates of cells in the VTA will increase in response to stimuli that are predictive of reward [39], local functional hyperconnectivity density in both the VTA and nucleus accumbens among cannabis users [26], and the results of our event‐related fMRI task that served as the cue‐exposure component of this study that reported increased fMRI activation in these regions in response to cannabis cues [24]. However, in the current experiment, we measured connectivity after the cues were no longer present and the expected reward was not delivered. It is possible that, because the visual and olfactory cues did not result in the expected reward, neural firing in the nucleus accumbens was transient and had decreased during the approximately 10‐min interval between the last exposure to cannabis cues and the end of the postCue resting state scan, which is the time period captured by our postCue resting state scan [39]. This interpretation aligns with the incentive salience theory of addiction, which posits that drug cues drive craving and motivational states but may not always result in prolonged changes in reward system connectivity when the reward is not obtained [1]. Further, it is possible our sample, composed of individuals at moderate risk for CUD, did not exhibit the same degree of dysregulation in VTA‐striatal circuitry that might be observed in individuals with more severe addiction. As such, in more severely affected individuals, chronic CU could lead to greater disruptions in dopaminergic signaling and connectivity between VTA and nucleus accumbens, reflecting a heightened sensitivity to drug‐related cues and a more pronounced neural adaptation to addiction. Future studies could investigate whether severity of CUD is a key moderator of VTA‐nucleus accumbens connectivity following cue exposure to help clarify the neurobiological mechanisms driving craving and relapse in CUD.

Exploratory whole brain analyses (see supplementary information) showed a significant group by cue‐exposure interaction effect in VTA–OFC connectivity. The OFC is involved in modulating the reinforcement value of a stimulus in the context of recent experience [40]. This finding is consistent with previous fMRI task‐based cue‐reactivity research that reported increased activation in the VTA and OFC in response to cannabis cues [41]. Notably, increased VTA activation was observed in frequent users of cannabis compared to sporadic users of cannabis, while increased OFC activation was only observed in frequent users with high problem severity, as measured by the CUDIT‐R, suggesting that the neurobiological changes in the VTA may precede the transition to problematic CU. In addition, we observed that the relationship between the post > pre resting state contrast and craving was characterized by increased connectivity with sensorimotor areas. In contrast to the striatum, which appears to respond to baseline self‐reported craving levels alone, this finding suggests that cue‐induced connectivity changes interact with the degree of craving in cortical networks that are closely tied to action readiness or motivational salience. Prior literature supports this interpretation: motor regions are consistently implicated in the planning and initiation of goal‐directed behavior, especially in the context of learned cue‐action associations (e.g., [42]). In addiction research, these areas are often activated during drug cue exposure and have been interpreted as reflecting preparatory motor responses or conditioned readiness to engage in drug‐seeking behaviour. Moreover, the incentive salience theory of addiction posits that drug‐related cues can trigger activation of motivational and motor systems independently of self‐reported desire, especially when these cues have acquired strong associative value [43]. Taken together, these findings suggest that cue‐elicited changes in motor‐related connectivity may reflect a generalized preparatory or motivational response rather than a direct neural correlate of momentary craving.

Finally, through our investigation of the relationship between craving and VTA‐striatal connectivity, we observed anticorrelations between the VTA, nucleus accumbens and head of the caudate, but positive correlations with the body/tail of the caudate and the putamen (see Figure 2). Note that we did not use global signal regression in our preprocessing pipeline, which can artificially induce anticorrelations in resting state data [44]. To our knowledge, anticorrelations in intrinsic connectivity between the VTA and nucleus accumbens have not been previously reported, and their physiological basis remains unclear given the complex neurocircuitry involved. The VTA releases dopamine, glutamine, GABA (excitatory and inhibitory neurotransmitters respectively) and also combinations of dopamine + GABA or glutamate via combinatorial neurons [45]. The connections between the VTA and the nucleus accumbens are largely dopaminergic but integrate both excitatory and inhibitory signalling neural pathways, which result in diverse output firing patterns that depend on the neurophysiological context [45]. Importantly, GABA‐ergic neurons directly project from the VTA to the nucleus accumbens and are associated with reward learning, while high CB_1_ receptor expressing GABA interneurons are likely responsible for acute disinhibition of dopamine release following acute THC exposure and reduced DA activity after chronic THC exposure and CB_1_ receptor downregulation [46]. This complexity may underlie the observed anticorrelations, which likely reflect dynamic shifts in the balance of inhibitory and excitatory signals modulated by craving. Although fMRI is an indirect measure of neural activity [47] and our connectivity measures cannot address causal directionality, our data indicate that the VTA and nucleus accumbens are primarily anticorrelated, but craving may induce neurophysiological changes in the balance between inhibitory and excitatory signalling. This process may be influenced by GABA and glutamate in addition to dopamine. In our fMRI data, the relationship between the VTA and NA connectivity transitions from anticorrelated to positively correlated neural signals with higher levels of self‐reported craving (see Figure 4, scatter plots). This shift may represent the dynamic influence of craving on reward processing, as heightened craving could drive the nucleus accumbens to prioritize excitatory signalling in response to reward‐related cues, reflecting enhanced motivational salience and the influence of cortical regions outside the mesostriatal pathway. It is plausible that other cognitive and/or physiological processes may also produce a similar pattern, although to our knowledge, none have been previously reported in the context of VTA‐striatal connectivity.

### 4.1 Limitations

While our results support inferences about the role of addiction neurobiology and cue responsivity in cannabis users, our study has several limitations that may impede its applicability to all cannabis users. First, our sample size was relatively small, which may limit statistical power and the generalizability of our preliminary findings. Second, the individual resting state scans we conducted were relatively short (6′40″ each) compared to current guidelines that suggest collecting 10–12 min of resting state data to improve reliability [48, 49]. Thus, our report of no significant between‐group differences for the standalone pre‐ and post‐cue resting state scans should be interpreted with extra caution. However, note that the primary outcomes (see Tables 2 and 3) highlighted in the discussion were based on analyses that combined both resting state scans, totalling 13′20″ of data. Third, despite the use of measures assessing CU and its impacts, including the CUDIT‐R, which may indicate risk for CUD, our study group was not clinically assessed for CUD and therefore, the prevalence of the clinical diagnosis is unknown in our study sample. Because of this limitation, the brain changes reported in this study could be associated with addiction or heavy use, limiting the interpretability of our results. Fourth, neuroimaging was performed 24–48 h after last CU, which for heavy cannabis users might be expected to be associated with symptoms of cannabis withdrawal and reversal of neuroadaptations associated with frequent CU [50]. While we did assess past withdrawal‐related experiences and found low rates of endorsement in this sample, the presence of withdrawal symptoms on the day of the scan were not captured and could have influenced our results. However, given the documented relationship between craving and withdrawal, co‐occurring symptoms, if present, would not necessarily undermine the relevance of our findings. Fifth, although we designed the study to target cannabis as the primary substance of use, participants in the CU group had significantly higher AUDIT and tobacco ASSIST scores. Further, we did not monitor alcohol and tobacco use on the day of the scan either through self‐report or using biological measures of use such as a breathalyser or urine drug screening. Study results did not change after controlling the AUDIT score, but statistical power was reduced (see Supplementary Information). In addition, potential tobacco addiction‐related impacts were not statistically controlled because of the floor effects on the ASSIST that were present in our sample (i.e., 32/49 participants scored 0). Because intoxication on the day of the scan was not assessed and differences in alcohol and tobacco use were not controlled through the experimental design, it remains possible that group differences in VTA‐striatal connectivity may have been influenced by these variables. Lastly, although significant changes in connectivity from pre‐ to post‐cue exposure were only observed in the cannabis group, because both drug cues and neutral cues were intermixed during the cue‐exposure component of this experiment, we cannot definitively conclude that the observed connectivity changes were driven specifically by the cannabis cues. Future studies with larger samples, formal diagnostic assessments, and control cue conditions are needed to replicate and extend these findings.

## 5 Conclusions

Drug cues have the power to induce drug use in an abstinent individual, which has led clinicians to advise patients to avoid the people, places and objects that are associated with their use [40]. Together, our preliminary findings support prior investigations demonstrating that alterations of mesocorticolimbic connectivity are associated with CU and craving in individuals at risk for CUD. In addition, our findings indicate that VTA‐caudate connectivity may have a particularly important role in predicting relapse, as altered connectivity persists after cannabis cues are no longer present.

## Author Contributions

NMK supervised this study. NMK, MEL and GET were responsible for funding acquisition and resources. Conceptualization of the study was done by NMK and MEL. Methodology was determined by NMK and MEL. NMK and MEL were responsible for project administration. Investigation, software, data curation and validation were done by NMK, GET, SFL, and DK. Formal analysis, visualization and writing of original draft were completed by NMK, GET, and MEL. All authors were involved in reviewing and editing the manuscript.

## Consent

Informed consent was obtained from all subjects involved in the study.

## Ethics Statement

This study was approved by the University of Washington Human Subjects Division Institutional Review Board.

## Conflicts of Interest

The authors declare no conflicts of interest.

## Supporting information


**Table S1.** Group differences in whole brain VTA connectivity.
**Figure S1.** Group × time interaction in ventral tegmental area (VTA)‐whole brain resting‐state connectivity. Significant clusters of activation with peak region in the medial orbital frontal cortex (left box) and the orbital frontal cortex (right box) are overlaid on the standard template brain, depicted in the centre of the image. Data are presented in radiological convention (R = L). The bar charts illustrate the mean z‐score of the all the voxels in the cluster at each time point for each group separately (controls = blue, CU = green). CU participants showed increased connectivity between the VTA and frontal regions, while the control participants did not show reduced VTA connectivity following cue exposure.
**Table S2.** Correlations between VTA connectivity and craving in cannabis users.
**Figure S2.** Correlations between cannabis craving and ventral tegmental area‐ connectivity changes following cue exposure and craving in the CU group. Positive mean cluster z‐scores indicate connectivity was stronger after the cue exposure compared to before. Top: Significant clusters of activation were observed in the right sensorimotor cortex (top right), right primary auditory cortex (top middle) and left sensorimotor cortex (top left). The significant cluster in the occipital cortex is not pictured. Bottom: Scatter plots reflect the mean z‐score within the significant cluster for each participant for the contrast postCue > preCue and are included for illustrative purposes only. Images are presented in radiological convention (R = L).
**Table S3.** Sensitivity analysis: Group differences in VTA‐striatal connectivity without the > 27 CU participant.
**Table S4.** Sensitivity analysis: Correlations (without > 27, *n* = 24) between VTA‐striatal connectivity and craving with chronic cannabis use.
**Table S5.** Sensitivity analysis: Group differences in VTA‐striatal connectivity, controlling for the AUDIT score.
**Table S6.** Sensitivity analysis: Correlations (*n* = 25) between VTA‐striatal connectivity and craving with chronic cannabis use, controlling for Age and AUDIT score.
**Figure S3.** This is an image of the ventral tegmental area (VTA) seed region used in our study overlaid on the Montreal Neurological Institute template brain. Images are presented in radiological convention (R = L). The VTA seed region was delineated using the Duke University Probabilistic Atlas of the Midbrain, using a 50% probability threshold. The heat map shows the probability of a given region being the VTA, with the darkest red indicating voxels with a 50% probability of being the VTA and the brightest yellow indicating a 100% probability of the voxel being part of the VTA.

**Data S1.** Quantification of Cannabis Consumption (QCC)

## Data Availability

The data that support the findings of this study are available on request from the corresponding author. The data are not publicly available due to privacy or ethical restrictions.
