# Supplementary material for: Increased Mesostriatal Intrinsic Connectivity Associated With Cue Exposure in Adult Cannabis Users: Preliminary Findings
Source: Addict Biol. 2025 Aug 7;30(8):e70067. doi: 10.1111/adb.70067 (PMC12331521; doi:10.1111/adb.70067)
Supplement: Supplementary file 1 — Table S1. Group differences in whole brain VTA connectivity. Figure S1. Group × time interaction in ventral tegmental area (VTA)‐whole brain resting‐state connectivity. Significant clusters of activation with peak region in the medial orbital frontal cortex (left box) and the orbital frontal cortex (right box) are overlaid on the standard template brain, depicted in the centre of the image. Data are presented in radiological convention (R = L). The bar charts illustrate the mean z‐score of the all the voxels in the cluster at each time point for each group separately (controls = blue, CU = green). CU participants showed increased connectivity between the VTA and frontal regions, while the control participants did not show reduced VTA connectivity following cue exposure. Table S2. Correlations between VTA connectivity and craving in cannabis users. Figure S2. Correlations between cannabis craving and ventral tegmental area‐ connectivity changes following cue exposure and craving in the CU group. Positive mean cluster z‐scores indicate connectivity was stronger after the cue exposure compared to before. Top: Significant clusters of activation were observed in the right sensorimotor cortex (top right), right primary auditory cortex (top middle) and left sensorimotor cortex (top left). The significant cluster in the occipital cortex is not pictured. Bottom: Scatter plots reflect the mean z‐score within the significant cluster for each participant for the contrast postCue > preCue and are included for illustrative purposes only. Images are presented in radiological convention (R = L). Table S3. Sensitivity analysis: Group differences in VTA‐striatal connectivity without the > 27 CU participant. Table S4. Sensitivity analysis: Correlations (without > 27, n = 24) between VTA‐striatal connectivity and craving with chronic cannabis use. Table S5. Sensitivity analysis: Group differences in VTA‐striatal connectivity, controlling for the AUDIT score. Table S6. Sensitivity analy [file ADB-30-e70067-s001.docx]

Whole Brain Analyses

Individual-level connectivity maps for the VTA were derived using FMRIB's Improved Linear Model (FILM) with local autocorrelation correction for each resting state scan. Within subject contrasts were computed using fixed effects analyses in FMRI Expert Analysis Tool (FEAT) for the following contrasts: preCue, postCue, and postCue>preCue. Positive correlations with the VTA timeseries were tested.

Higher level analyses of between-group differences for all contrast (preCue, postCue, and postCue>preCue) were conducted using FLAME (FMRIB’s Local Analysis of Mixed Effects). Statistical corrections for multiple comparisons were conducted using cluster-thresholding based on Markov Chain Monte Carlo sampling (voxel height of z > 2.3) and a whole brain corrected cluster significance threshold of p < .05).

To measure correlations between VTA connectivity changes related to cue exposure and cannabis craving in the CU group, the MCQ-SF total scores were entered as regressors, controlling for age, for the contrast postCue>preCue.

Between Group Comparisons

There were no significant between-group differences in VTA-striatal connectivity during the preCue or postCue scan.

Interaction between groups and cue exposure

There was a significant group by cue interaction effect in the medial and orbital frontal cortex. (Table S1, Figure S1). The control participants demonstrated reduced connectivity between the pre and postCue resting state scans, while the CU group showed increased connectivity at postCue relative to the preCue resting-state scan.

Correlations between connectivity and cannabis craving

Higher levels of self-reported craving were significantly associated with increased connectivity between the VTA in primary sensorimotor cortex, primary auditory cortex, and the lateral occipital cortex following cue exposure. No significant negative correlations were observed. See Table S2, Figure S2.

Figure S1. Group x time interaction in ventral tegmental area (VTA)-whole brain resting-state connectivity. Significant clusters of activation with peak region in the medial orbital frontal cortex (left box) and the orbital frontal cortex (right box) are overlaid on the standard template brain, depicted in the center of the image. Data are presented in radiological convention (R=L). The bar charts illustrate the mean z-score of the all the voxels in the cluster at each time point for each group separately (controls = blue, CU = green). CU participants showed increased connectivity between the VTA and frontal regions, while the control participants did not show reduced VTA connectivity following cue exposure.
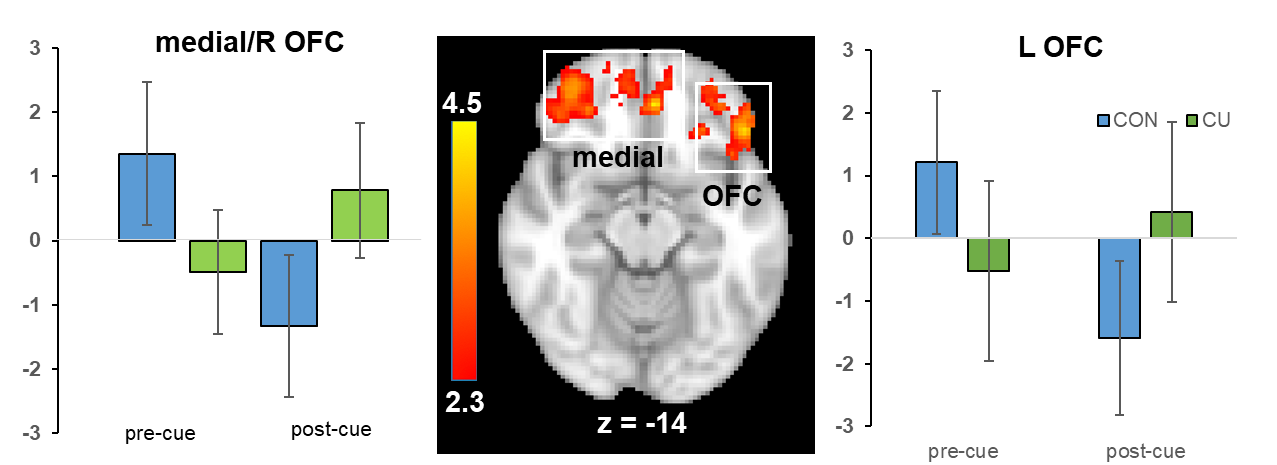

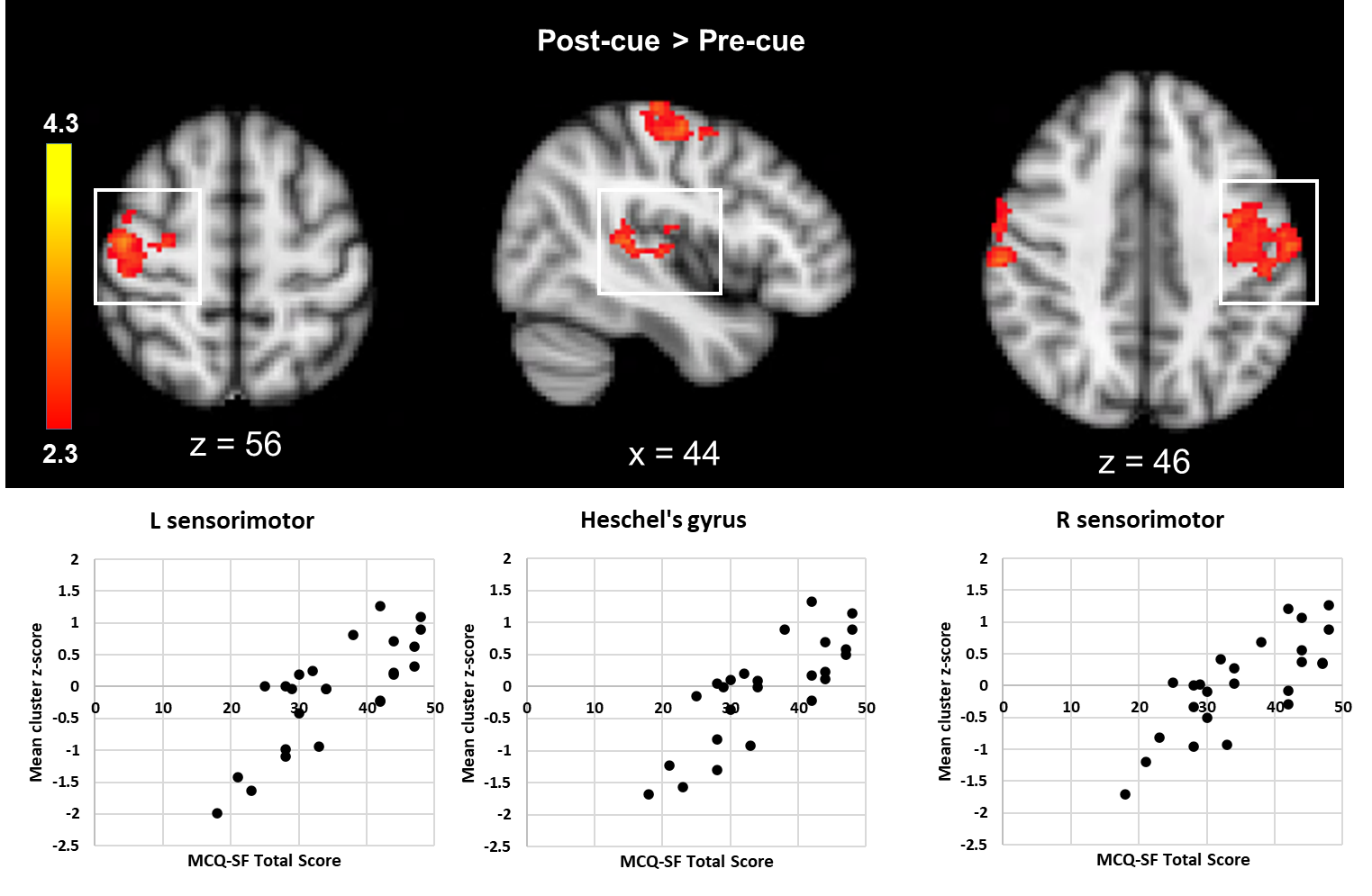
Figure S2. Correlations between cannabis craving and ventral tegmental area- connectivity changes following cue exposure and craving in the CU group. Positive mean cluster z-scores indicate connectivity was stronger after the cue exposure compared to before. Top: Significant clusters of activation were observed in the right sensorimotor cortex (top right), right primary auditory cortex (top middle), and left sensorimotor cortex (top left). The significant cluster in the occipital cortex is not pictured. Bottom: Scatter plots reflect the mean z-score within the significant cluster for each participant for the contrast postCue>preCue and are included for illustrative purposes only. Images are presented in radiological convention (R=L).

Table S3. Sensitivity analysis: Group differences in VTA-striatal connectivity without the > 27 CU participant

|  |  |  |  |  |  |  | **MNI (mm)** | | |
| --- | --- | --- | --- | --- | --- | --- | --- | --- | --- |
| **Contrast** | | **+/- corr** | **ROI** | **voxels** | **p value** | **z-max** | **x** | **y** | **z** |
| **postCue>preCue** | | | | | | | | | |
|  | CU > CON | + | Right caudate | 88 | .027 | 3.63 | 14 | 22 | 6 |
| Note. Regions were delineated using the Oxford-Imanova Striatal Structural Atlas. P-values are based on small volume cluster correction for multiple comparisons. Acc= accumbens. | | | | | | | | | |

Table S4. Sensitivity analysis: Correlations (without >27, n=24) between VTA -striatal connectivity and craving with chronic cannabis use.

|  |  |  |  |  |  | **MNI (mm)** | | |
| --- | --- | --- | --- | --- | --- | --- | --- | --- |
| **Contrast** | **+/- corr.** | **ROI** | **voxels** | **p value** | **z-max** | **x** | **y** | **z** |
| **preCue +postCue** | + | Right nucleus acc. | 104 | .008 | 4.12 | 8 | 8 | -2 |
|  | + | Left nucleus acc. | 63 | .017 | 4.07 | -14 | 20 | -8 |
|  | + | Right putamen | 377 | .001 | 3.99 | 26 | 8 | -6 |
|  | + | Left caudate | 130 | .011 | 3.46 | -12 | 14 | -4 |
|  | + | Right caudate | 53 | .057 | 4.08 | 14 | 18 | -2 |
| Note. Regions were delineated using the Oxford-Imanova Striatal Structural Atlas. P-values are based on small volume cluster correction for multiple comparisons. Acc= accumbens. Correlational analyses included age a covariate. | | | | | | | | |

Table S5. Sensitivity analysis: Group differences in VTA-striatal connectivity, controlling for the AUDIT score

|  | |  |  |  |  |  |  | **MNI (mm)** | | |
| --- | --- | --- | --- | --- | --- | --- | --- | --- | --- | --- |
| **Contrast** | | | **+/- corr** | **ROI** | **voxels** | **p value** | **z-max** | **x** | **y** | **z** |
| **PostCue > PreCue** | | | | | | | | | | |
|  | CU (n=25) | | + | Left caudate | 77 | .033 | 3.22 | -18 | 18 | 6 |
|  | CU (n=25) > CON | | + | Right caudate | 125 | .013 | 3.57 | 16 | 18 | 14 |
|  |  | |  | Left caudate | 81 | .031 | 3.57 | -18 | 18 | 6 |
| Note. Regions are labeled using the Oxford-Imanova Striatal Structural Atlas. P-values are based on small volume cluster correction for multiple comparisons. | | | | | | | | | | |

Table S6. Sensitivity analysis: Correlations (n=25) between VTA-striatal connectivity and craving with chronic cannabis use, controlling for Age and AUDIT score

|  |  |  |  |  |  | | **MNI (mm)** | | |
| --- | --- | --- | --- | --- | --- | --- | --- | --- | --- |
| **Contrast** | **+/- corr.** | **ROI** | **voxels** | **p value** | **z-max** | | **x** | **y** | **z** |
| **PreCue +PostCue** | + | Right nucleus acc. | 79 | .012 | 4.03 | 8 | | 8 | -2 |
|  | + | Left nucleus acc. | 54 | .021 | 3.91 | -12 | | 18 | -8 |
|  | + | Right putamen | 357 | .001 | 3.89 | | 28 | 10 | 0 |
|  | + | Left caudate | 121 | .016 | 3.44 | | -16 | 22 | -4 |
|  | + | Right caudate | 61 | .048 | 3.84 | | 14 | 18 | -2 |
| Note. Regions were delineated using the Oxford-Imanova Striatal Structural Atlas. P-values are based on small volume cluster correction for multiple comparisons. Acc= accumbens. | | | | | | | | | |


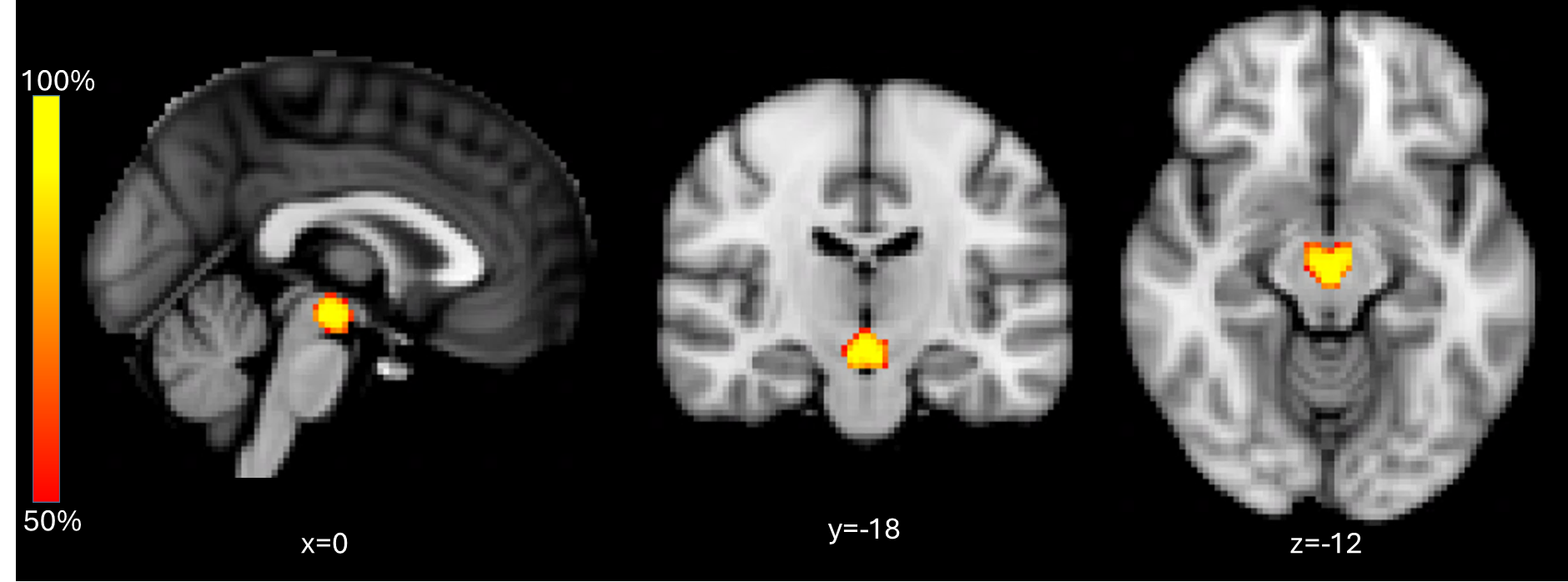


Figure S3. This is an image of the ventral tegmental area (VTA) seed region used in our study overlaid on the Montreal Neurological Institute template brain. Images are presented in radiological convention (R=L). The VTA seed region was delineated using the Duke University Probabilistic Atlas of the Midbrain, using a 50% probability threshold. The heat map shows the probability of a given region being the VTA, with the darkest red indicating voxels with a 50% probability of being the VTA and the brightest yellow indicating a 100% probability of the voxel being part of the VTA.
