# Supplementary material for: Increased Mesostriatal Intrinsic Connectivity Associated With Cue Exposure in Adult Cannabis Users: Preliminary Findings
Source: Addict Biol. 2025 Aug 7;30(8):e70067. doi: 10.1111/adb.70067 (PMC12331521; doi:10.1111/adb.70067)
Supplement: Supplementary file 2 — Data S1. Quantification of Cannabis Consumption (QCC) [file ADB-30-e70067-s002.docx]

**Quantification of Cannabis Consumption (QCC)**

We are interested in understanding the biologic basis of how marijuana use affects brain function. Your responses on the following questions will be used for this purpose and will be kept confidential.

Although marijuana is often referred to by many names (cannabis, pot, weed, etc.), we will refer to it as marijuana in this survey.

1. In your lifetime, how many times have you used marijuana:

Never (Stop here – this form is done.)

1-5 times

6-15 times

More than 15 times

1. How old were you when you first used marijuana? ____________
2. For how long, altogether, have you used marijuana at least once a week or more?

_________ months _________ years

1. During what ages did you use marijuana once a week or more? (check all that apply)

0 – 10

10 – 15

16 – 20

21 – 25

26 – 30

30+

1. Have you ever had a bad reaction to marijuana, such as becoming very frightened, paranoid or upset?

No

Yes

Kind of or something else:

1. If you have stopped or significantly reduced your use of marijuana, what was the motivation/reason?

I’ve reduced my use, because of

I’ve stopped using, because of

*If you have used marijuana within the past 3 months, please continue to the next page*

*If your typical use has changed lately, focus on the last 30 days.*

1. How often do you typically use marijuana?

Monthly or less

2-4 times per month

2-3 times per week

4 or more times per week

1-2 times a day

3-4 times per day

4 or more times per day

1. Please select any form(s) of marijuana you commonly consume, and answer the corresponding questions about method and amount of use.

| *What form do you use?* | *How do you consume it?* | *How much do you use?* |
| --- | --- | --- |
| Bud  Hash | rolled joint  blunt  spliff  pipe  bong/water  vaporizer | On a typical day of use, how many grams do you use?  ______________ grams |
| Oil  Dab/Wax/Shatter  Kief (crystals) | vaporizer (e.g., a pen)  dab/oil rig | How long does ½ gram last you?  _______________________________ |
| Tincture  Edibles | swallowed  under the tongue  topical/skin | On a typical day of use, how many milligrams (mg) would you consume of:  THC ___________________________  CBD ___________________________ |

*If you have an alternative or more specific explanation of how you consume marijuana, please describe:*

1. What strain of marijuana do you typically use?

Sativa

Indica

No preference

Sativa dominant hybrid

Indica dominant hybrid

Not sure

1. If you purchase marijuana for yourself, is there a name brand you most commonly buy? (for example, ‘sour diesel’)

1. When you use marijuana, what is the intended effect? (Check all that apply)

Recreational / to feel good

Feel ‘stoned’

Decrease nightmares

Improve sleep problems (other than nightmares)

Decrease pain

Decrease anger / irritability

Forget my problems for a while

Be social

Decrease anxiety / stress

Improve depression / sadness

Increase energy

Other:____________________

1. When do you typically use marijuana? (Check all that apply)

First thing in the morning

Throughout the day

Evening

Before bed

Weekends only

Other:___________________________

1. Have you ever noticed… (Check all that apply)

Needing to use more marijuana than you used to in order to get the same effect?

Mood symptoms, such as irritability, anxiety, or depressed mood, when you stop or decrease your use?

Physical symptoms such as shakiness, abdominal pain, sweatiness, or headache when you stop or decrease your use?

Sleep problems when you stop or decrease your use?

Changes in appetite when stop or decrease your use?

1. Do you use marijuana *instead of or to replace* other substances? (Check all that apply)

Tobacco

Alcohol

Spice/K2/Synthetic marijuana

Prescription opiates

Other medications: ________________

Cocaine

Methamphetamine

Heroin

Over the counter medications

Other: __________________________

1. Overall, my use of marijuana has had….

A positive impact on my life

A negative impact on my life

No significant impact on my life either way

Both positive and negative impacts on my life
